# Long-term effects of rootstock and tree density on the economic profitability of ‘Delicious’ apple orchards in the Northeastern U.S

**DOI:** 10.3389/fpls.2026.1762635

**Published:** 2026-03-03

**Authors:** Luis Gonzalez Nieto, Stephen A. Hoying, Gemma Reig, Jaume Lordan, Poliana Francescatto, Michael J. Fargione, Gennaro Fazio, Terence L. Robinson

**Affiliations:** 1 School of Integrative Plant Sciences, Horticulture Section, New York State Agricultural Experiment Station, Cornell University, Geneva, NY, United States; 2 Fruit Production Programme, Institute of Agrifood Research and Technology (IRTA), Lleida, Catalonia, Spain; 3 Research Group in AgroICT & Precision Agriculture, University of Lleida, Lleida, Catalonia, Spain; 4 Agrotecnio-CERCA Center, Lleida, Catalonia, Spain; 5 Valent BioSciences, Libertyville, IL, United States; 6 Ulster County Cooperative Extension, Hudson Valley Research Laboratory, Highland NY, United States; 7 U.S. Department of Agriculture's (USDA) Agricultural Research Service (ARS) Plant Genetic Resources Unit, New York State Agricultural Experiment Station, Geneva, NY, United States

**Keywords:** net present value, orchard design, orchard profit, planting density, sustainability, yield

## Abstract

Establishing an apple orchard involves a strategic combination of biological and structural decisions. Factors such as variety, rootstock, tree spacing, training system, and local environmental and economic conditions all interact to influence orchard performance over time. Understanding how these variables affect long-term profitability is essential for growers aiming to maximize returns on investment. This study presents an economic evaluation of a long-term field trial conducted in New York State (Yonder Farm, southeastern region) from 2007 to 2017. The trial focused on ‘Delicious’ apples and assessed the interaction between ten rootstocks (B.118, G.11, G.16, G.210, G.30, G.41, G.935, M.26, M.7, and M.9) and four training systems with varying planting densities: Super Spindle (5,382 trees·ha^-1^), Tall Spindle (3,662 trees·ha^-1^), Triple Axis Spindle (2,243 trees·ha^-1^), and Vertical Axis (1,656 trees·ha^-1^). Our results show that high-density systems, Super Spindle and Tall Spindle, consistently delivered the highest profitability, despite their higher initial establishment costs. These systems also achieved faster break-even points and greater cumulative net present value, especially with rootstocks such as G.11, G.210, and G.935. In contrast, lower-density systems like Vertical Axis and Triple Axis Spindle showed slower economic recovery and lower overall returns. The multi-leader Triple Axis Spindle system had lower profitability than higher density single stem systems (Tall Spindle and Super Spindle). This indicates that multi-leader trees planted at lower planting densities than Tall Spindle or Super Spindle with the goal to reduce initial establishment costs does not result in as high profitability as the higher density single stem systems. Profitability was not only influenced by training system but also by the compatibility between rootstock and planting density. Rootstocks such as G.41, G.11, and G.210 performed best under high-density conditions, while B.118 was more suited to low-density systems. Conversely, M.9 and M.7 showed limited economic potential, particularly when used in intensive planting systems. These findings underscore the importance of aligning rootstock vigor and precocity with the structural design of the orchard to optimize long-term economic outcomes.

## 1 Introduction

The investment required to establish a new apple orchard is one of the most important factors in lifetime profitability. The decisions made at planting time have a large influence on the future profitability of the orchard. Thus, design phase of the orchard is critical in the long-term profitability with important decisions such as variety, planting density, training system and rootstock all having a large effect. Previous economics analyses showed that with different apple varieties the selection of the rootstock (Gonzalez Nieto et al., 2024; Lawrence et al., 2025) and the training system were the most important factors in the final profitability of the orchard (Gonzalez Nieto et al., 2023; Ho et al., 2024). Several studies of horticultural and economic performance of new orchards showed that training system and rootstock can improve yield and profitability of the orchard (Gonzalez Nieto et al., 2023; Lordan et al., 2019a, 2018a, 2019b; Reig et al., 2020, 2019; Robinson, 2007; Robinson et al., 2007). There are several studies that evaluated mineral nutrient profiles of different rootstocks (Reig et al., 2018; Fazio et al., 2018, 2019, 2025), the effect of rootstocks on fruit disorders (Donahue et al., 2021; Islam et al., 2022; Valverdi and Kalcsits, 2021) or resistance to different apple pest (Kviklys et al., 2016; Leinfelder and Merwin, 2006; Norelli et al., 2003; Russo et al., 2007), hormone concentrations (Kalcsits et al., 2019; Lordan et al., 2017; Van der Krieken et al., 1993) and organic management (Bradshaw et al., 2015; Fazio et al., 2025). However, all these articles evaluated agronomical parameters and not the long-term economic implications. One exception was Lordan et al. (2019b) that evaluated the economic effects in two cultivars at different tree densities and five rootstocks. They observed both high and medium planting density systems and found that M.9 and B.9 offer a more favorable balance between tree vigor, productivity, and profitability than more vigorous rootstocks. Another study by Ho et al. (2024) evaluated eleven rootstocks in four training systems at different planting densities and three cultivars in two locations. They found that the most profitable training system was Tall Spindle at 3230 trees per ha with the best rootstocks being G.11, G.16, G.41, M.9 and B.9. in ‘Gala’, ‘Honeycrisp’ and ‘Fuji’. However, there are no economic studies specifically focused on the ‘Delicious’ cultivar, particularly evaluating the interaction between multiple rootstocks and training systems over the long term, which represents a significant gap in the literature that our study aims to address.

‘Delicious’ is one of the most important varieties in USA and in other areas of the world. In 2024 the USA production was 789,918 t (41.4 million bushels) and was the second most produced variety (Industry Outlook report 2025, www.usapple.org). The production of New York was 43,260 t (2 million bushels) and is one of the top 10 varieties in the state (Industry Outlook report 2025, www.usapple.org). We initiated this project to study the economics new orchards of this important variety since its spur-type clones have a very different growth habit than other varieties such as ‘Gala’, ‘Honeycrisp’ and ‘Fuji’ which we have already studied.

Among planting systems which we have not previously evaluated long-term economic performance is the multi-leader system. In the current study we chose to evaluate a triple leader system we named the triple axis spindle (TAS). Muli-leader systems of 2, 3 or more leaders have been proposed as a method of obtaining the desired number of leaders (trunks) per hectare with fewer trees per hectare thus reducing the initial establishment costs of high density orchards. The spur-type growth habit of ‘Delicious’ is optimum for such multiple leaders which can be spaced every 40 cm along the row. In this study we compared the TAS to both lower and higher density systems which utilized a single leader per tree. We also evaluated each system on a number of rootstocks to evaluate the interaction of rootstock and planting system. Building on this foundation, our objective was to evaluate the long-term economic performance and the interaction between ten different rootstocks and four training systems using a popular spur-type clone of ‘Delicious’: Super Chief. The horticultural results of this project were previously published by Reig et al. (2020), providing critical insights into tree growth and productivity. By extending this work to include an economic analysis, the present study offers practical guidance for optimizing orchard profitability and sustainability, which is essential for apple production systems in the Northeastern United States.

## 2 Materials and methods

### 2.1 Site description and experimental design

In 2007, an orchard experiment covering 0.8 hectares was set up at Yonder Farm, located in the Hudson Valley in the southeastern region of New York State (42°26’33.1”N 73°41’24.5”W at 95 meters above sea level). The study involved a comparison of ten rootstocks and four training systems, each representing a different planting density, using *Malus domestica* Borkh. ‘Delicious’ cultivar, Super Chief strain (spur-type clone). Among the rootstocks, four were traditional types used as controls, while six belonged to the Geneva^®^ series. The Geneva^®^ rootstocks tested were G.11 (Dwarf), G.16 (Dwarf), G.30 (Semi dwarf), G.41 (Dwarf), G.210 (Semi dwarf), and G.935 (Dwarf). The control rootstocks were B.118 (Semi dwarf), M.7EMLA (M.7) (Semi dwarf), M.9T337 (M.9) (Dwarf), and M.26EMLA (M.26) (Dwarf), as shown in **Table 1**.

**Table 1:** Training systems, spacings and rootstocks evaluated at the experimental trial in NY State.

System	Spacing and planting density	Rootstocks
Super Spindle	0.62 m × 3.00 m, 5382 trees•ha^-1^	B.118, G.11, G.16, G.210, G.30, G.41, G.935, M.26, M.7, M.9
Tall Spindle	0.92 m × 3.40 m, 3662 trees•ha^-1^	B.118, G.11, G.16, G.210, G.30, G.41, G.935, M.26, M.7, M.9
Triple Axis Spindle	1.22 m × 3.70 m, 2243 trees•ha^-1^	B.118, G.11, G.16, G.210, G.30, G.41, G.935, M.26, M.7, M.9
Vertical Axis	1.51 m × 4.00 m, 1656 trees•ha^-1^	B.118, G.11, G.16, G.210, G.30, G.41, G.935, M.26, M.7, M.9

The training systems evaluated in the trial included Super Spindle (SS), Tall Spindle (TS), Triple Axis Spindle (TAS), and Vertical Axis (VA), which are also listed in **Table 1**. Each training system was planted using the standard tree spacing recommended in New York for these systems. The range of densities covered by the four planting systems allowed for an evaluation of the effect of planting density on profitability. Detailed information regarding tree management practices, block arrangement, and soil characteristics was previously published by Reig et al. (2020).

The experimental setup followed a split-plot randomized complete block design with three replications. In each block, the main plot was the training system, while the rootstock genotype was the subplot. Treatments were arranged in a full factorial design, resulting in 40 combinations (4 training systems × 10 rootstocks). All trees used in the study were standard non-feathered nursery trees that had undergone two years of nursery cultivation.

Trees were irrigated using drip lines. VA trees were supported by a steel conduit pipe attached to a single-wire trellis, whereas SS, TA, and TAS trees were supported by a three-wire trellis system. Details regarding pruning, thinning, irrigation, fertilization, foliar micronutrient applications, and pest management practices were provided in Reig et al. (2020). The average annual rainfall at Yonder farm site was approximately 1000 mm, primarily occurring during the spring and summer seasons.

### 2.2 Yield, income and costs

Tree horticultural performance was assessed over an eleven-year period, from 2007 to 2017, following planting. Starting in the second year, 2008, annual measurements were taken for yield (kg) and total fruit count. The average fruit size, expressed as weight, was calculated based on the total yield and number of fruits. Each year at harvest, a sample of 50 apples representing each rootstock and training system combination was collected from each replicate. These samples were then classified by color and size according to the methodology described by Reig et al. (2020). Using this classification data, a simulated packout was generated for each rootstock and training system combination. Monetary values were assigned to each fruit size and quality category based on statewide average market prices from the New York apple industry in 2024, as shown in **Table 2**. The economic value of each category was summed to calculate the crop value per tree and per hectare. These values were then used to perform the economic analysis.

**Table 2:** Returns to apple growers by grade, and fruit size, after subtracting storage and packing costs for ‘Delicious’ apples.

Color category	Grower returns ($/kg)								
	Fruit size (g)								
	<128	128 <136	136 <153	153 <167	167 <190	190 <215	215 <238	238 <264	≥ 264
XX Fancy	0.09	0.17	0.58	0.58	0.58	0.68	0.79	0.89	0.89
X Fancy	0.09	0.17	0.52	0.52	0.52	0.63	0.73	0.84	0.84
Fancy	0.09	0.17	0.42	0.42	0.42	0.52	0.52	0.52	0.52
No. 1	0.09	0.17	0.17	0.17	0.16	0.17	0.17	0.17	0.17
Utility	0.09	0.17	0.17	0.17	0.17	0.17	0.17	0.17	0.17

These costs included packing charge, MCP (1-methylcyclopropene) treatment, and average cost between regular and CA storage. Values were taken from statewide averages of New York State apple industry.

The tree price used in the analysis was based on the published price list for apple trees from a commercial nursery located in New York State (Wafler Nursery, 2024). Labor time for pruning was recorded annually, and average values were applied in years when data was unavailable. Pruning and training costs were calculated using a skilled labor rate of $15 per hour. Harvest costs were derived from statewide averages and estimated at $0.08 per kilogram, as shown in **Table 3**. Fixed costs also included management labor by the owner or manager, along with overhead expenses related to general farm operations (**Table 3**). Additional costs for pest control, disease management, weed control, fertilization, and chemical thinning were obtained from statewide averages reported by New York State apple growers (**Table 4**).

**Table 3:** Costs and parameters used in the economic analysis for ‘Delicious’ apple.

Pre-plant cost	
Land Value ($/ha)	$12,500
Land Preparation ($/ha)	$1,800
Labor: Planting, Training ($/ha)	$ 900
Tree price ($/Tree)	
Traditional rootstock (B and M clones)	$8.00
Geneva^®^ clones	$9.00
Trellising	
Trellis material ($/ha)	
Super Spindle	$16,688
Tall Spindle	$12,515
Triple Axis Spindle	$10,342
Vertical Axis	$7,507
Miscellaneous	
Irrigation Material ($/ha)	$2,500
Irrigation Install Labor ($/ha)	$1,000
Financials	
Interest rate (Discount rate)	5%
Annual fixed cost ($/ha)	$1,500
Wage rate ($/hour)	
Skilled labor	$15/h
Unskilled labor	$12/h
Picking	
Base Picking Cost ($/Bin)	$24
Picking Employer Taxes %	15%
Total Picking Cost ($/Bin)	$28
Total Picking Cost ($/kg)	$0.08

The list of costs is a proprietary non-published list of costs developed by Terence Robinson and is based on discussions with extension economists, growers, marketers and equipment suppliers.

**Table 4:** Annual costs for IPM, fertilizer applications, and chemical thinning for ‘Delicious’ apple, over a 20-year orchard life.

Year	N° year	IPM ($/ha)			Nutrition ($/ha)	Thinning ($/ha)
		Disease	Weed	Insects		
2005	0	0	47	0	558	0
2006	1	0	47	0	558	0
2007	2	252	79	106	850	0
2008	3	346	84	105	205	0
2009	4	591	25	345	432	39
2010	5	638	86	558	353	213
2011	6	717	126	661	610	338
2012	7	600	42	808	413	338
2013	8	581	86	625	492	338
2014	9	729	124	463	531	338
2015	10	729	124	632	492	338
2016	11	729	124	632	413	338
2017	12	729	124	632	489	338
2018	13	729	124	632	489	338
2019	14	729	124	632	489	338
2020	15	729	124	632	489	338
2021	16	729	124	632	489	338
2022	17	729	124	632	489	338
2023	18	729	124	632	489	338
2024	19	729	124	632	489	338
2025	20	729	124	632	489	338

Values were estimated from statewide averages of New York State apple growers. We used average data from year 8–11 to estimate values for years 12- 20.

### 2.3 Economic analysis

Net crop revenue was determined by subtracting storage and packing-related expenses from the gross crop revenue. Annual profit for each year was then calculated by deducting all costs from the net crop revenue. These annual profits were discounted using the Net Present Value (NPV) method to evaluate the performance of each training system and rootstock over a 20-year period. This analysis included the pre-planting year (2006) and extended from the planting year in 2007 through 2026.

Cash returns for the years 2017 to 2025 were projected based on actual harvest quantities and market prices recorded between 2014 and 2017. For example, the return for 2018 was estimated as the average of returns from 2014 to 2017, and returns for subsequent years were calculated using a moving average based on the same approach.

The economic analysis accounted for the time value of money by applying discounted annual cash flows, recognizing that money received today holds more value than the same amount received in the future. NPV was calculated as the sum of these discounted cash flows over the 20-year period, using a fixed discount rate. This rate was derived by subtracting the inflation rate from the current interest rate to obtain a real interest rate. A discount rate of 5% was applied for baseline comparisons, consistent with the methodology used by Lordan et al. (2018b) and Lordan et al. (2019b).

According to the NPV analysis, if the accumulated discounted profit yields a value greater than zero, the investment is considered to generate a positive long-term return at the selected discount rate (White and DeMarree, 1992). The year in which the accumulated NPV reaches zero marks the point at which the investment has been fully recovered with interest, referred to as the “break-even year” in the analysis presented below.

### 2.4 Statistical analysis

Data was analyzed using a randomized complete block split-plot design to evaluate the influence of rootstock and training system on the 20-year NPV. Cumulative 20-year NPV values were assessed through analysis of variance (ANOVA) using the Proc GLM procedure in SAS 9.4 (SAS Institute Inc., 2009, Cary, NC, USA). Mean comparisons were performed using Fisher’s Least Significant Difference (LSD) test at a significance level of *P* = 0.05, through one-way or factorial ANOVA, considering rootstock and training system as the main factors. When a significant interaction between rootstock and training system was detected, LSD values were calculated separately for each training system. Regression analysis was conducted to explore the relationship between final NPV and planting density throughout the orchard’s lifespan, using JMP13 statistical software (SAS Institute).

## 3 Results

### 3.1 Net revenue effects

**Figure 1:**
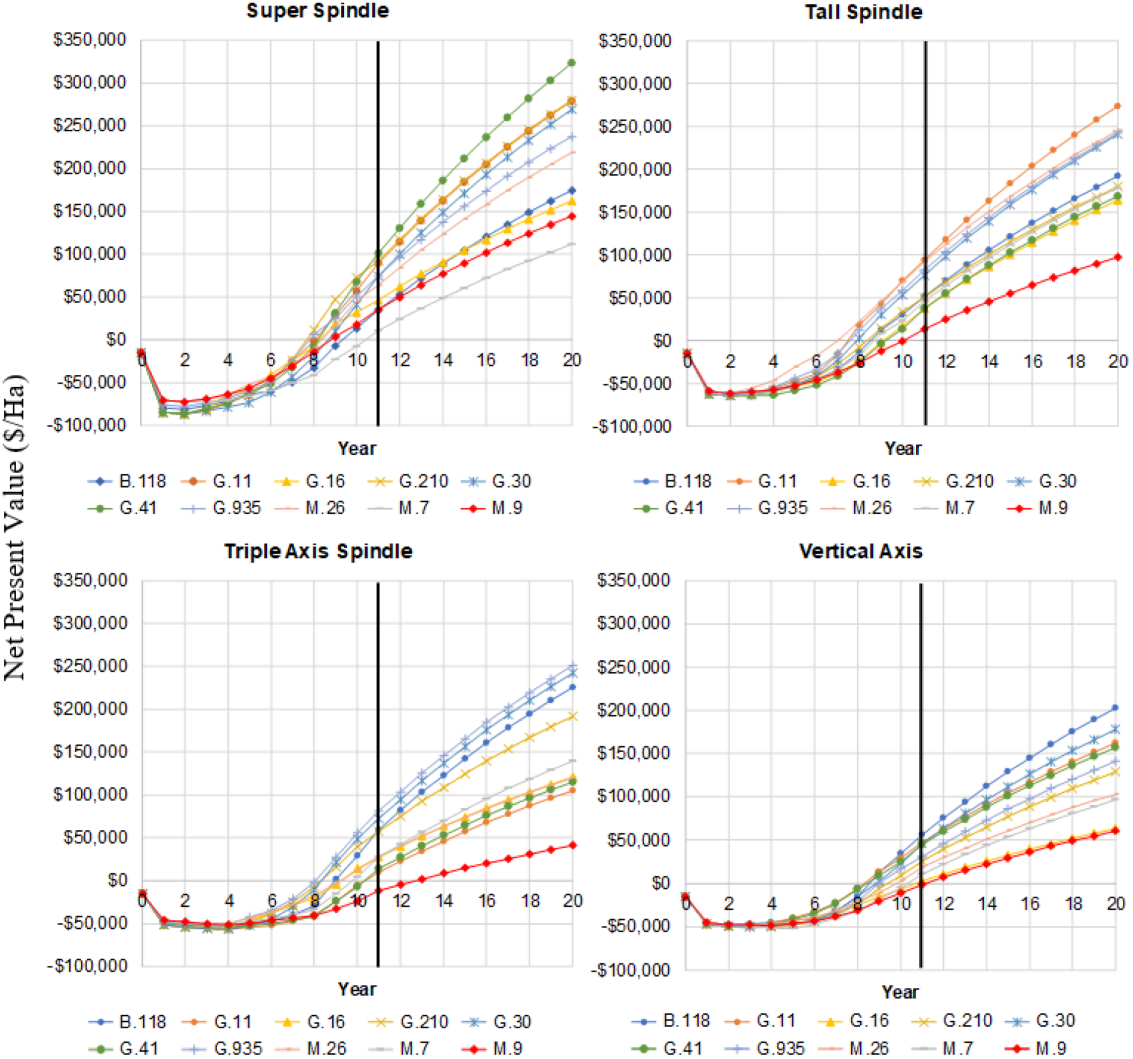
Cumulative NPV of net cash returns for ‘Delicious’ under various rootstocks and training systems in southeastern New York state. Vertical black line indicates that data from planting through year 11 was the actual field observed data while data after year 11 was estimated based on the final 3 years of field data.

**Figure 2:**
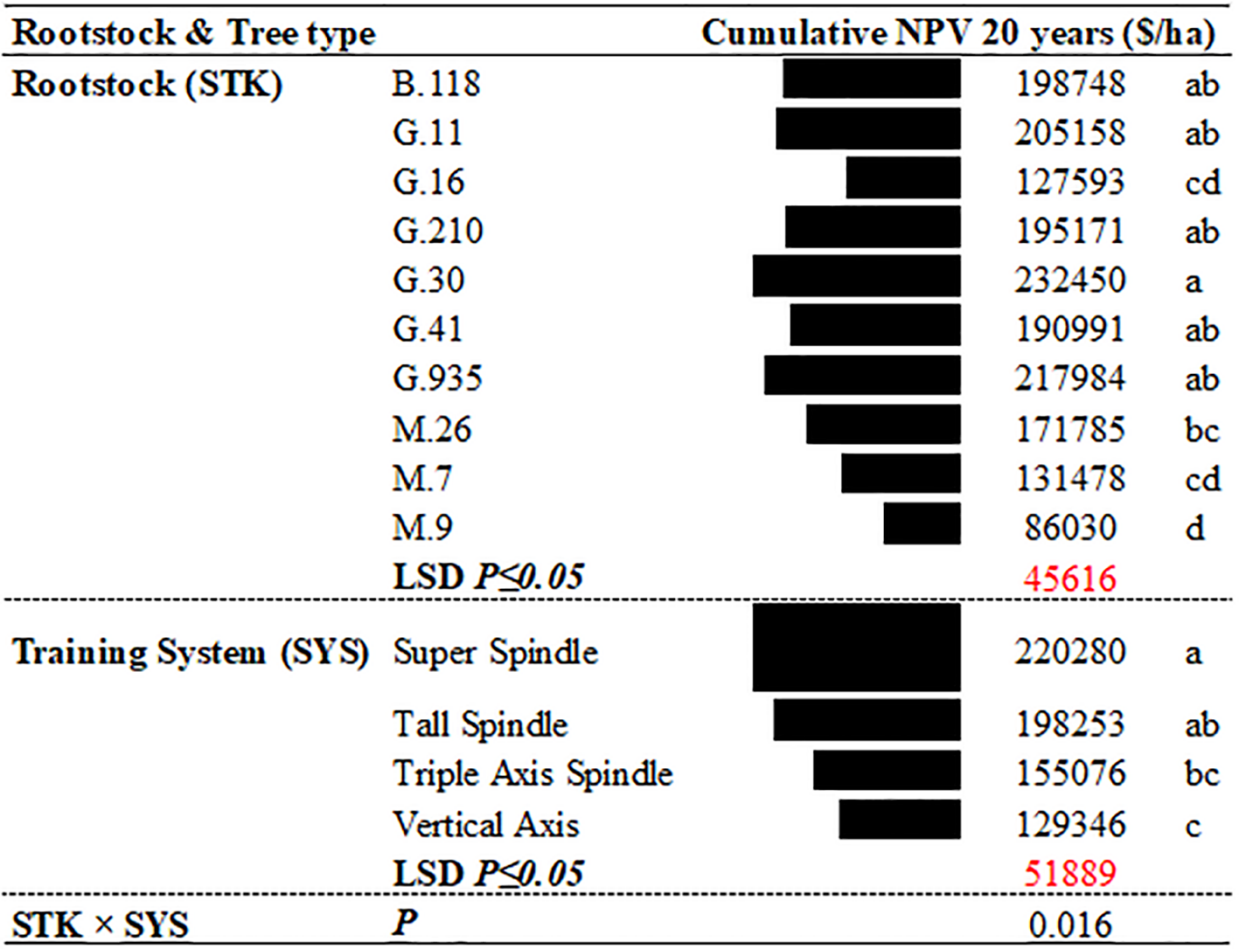
Main effects of rootstock genotype and training system on estimated NPV of 20-year net cash returns of ‘Delicious’ in southeastern New York State.

**Figure 3:**
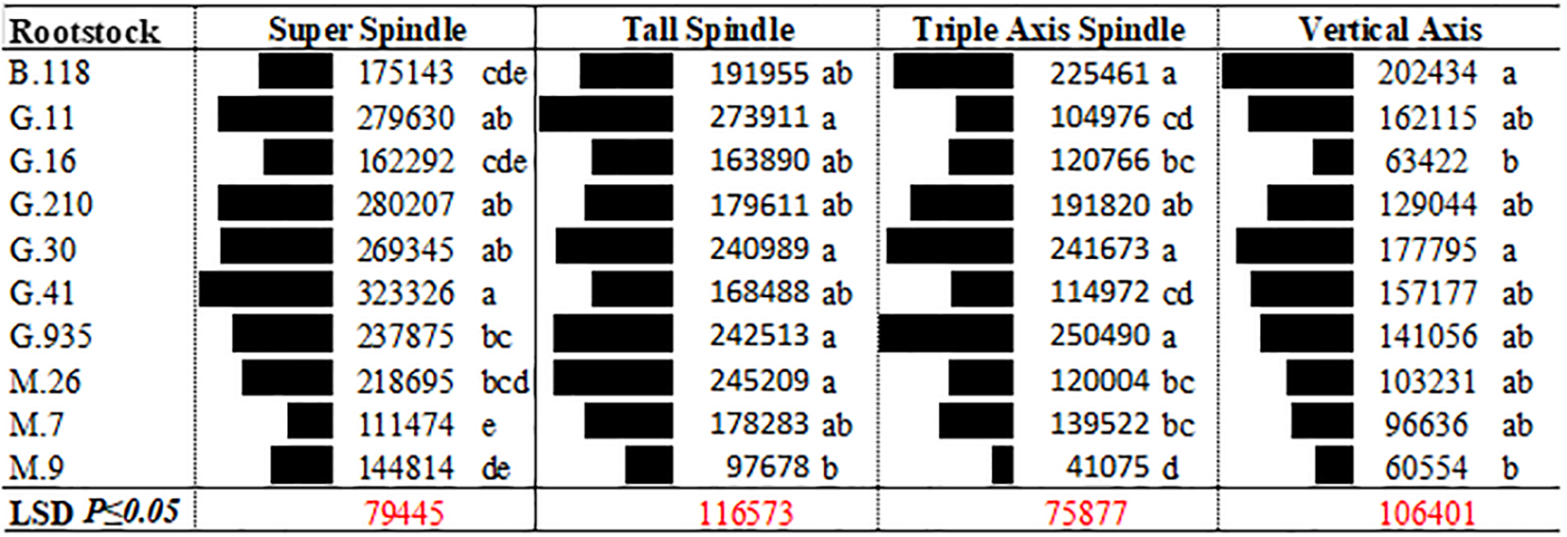
Interaction of rootstock genotype and training system on estimated NPV of 20-year net cash returns of ‘Delicious’ in southeastern New York State.

Both training system and rootstock had significant effects on the 20-year NPV (**Figures 1**–**3**). Among the training systems evaluated, SS and TS consistently resulted in the highest profitability, followed by TAS, while the VA system showed the lowest economic returns (**Figure 3**). Among rootstocks, G.30 provided the highest economic return over the 20-year period. However, its performance was not statistically different from that of G.935, G.11, B.118, G.210, and G.41, which also demonstrated high profitability. These rootstocks formed a group of top performers in terms of long-term NPV, indicating their strong potential for economic sustainability in apple production systems. In contrast, the remaining rootstocks showed significantly lower NPV values (M.26, M.7, G.16 and M.9), suggesting reduced profitability over the same period (**Figure 3**). However, significant interaction between training system and rootstock was also observed, suggesting that the economic performance of each rootstock varied depending on the training system. This highlights the importance of considering the compatibility between rootstock and training system when aiming to optimize orchard profitability.

Across most training systems, the rootstock M.9 was associated with the lowest NPV, except for the SS system, where M.7 had the poorest performance (**Figures 1**-**3**). In contrast, within the SS system, G.41 and G.11 emerged as the most profitable rootstocks. For the TS system, G.11 was the top performer, followed by M.26, G.935, and G.30. In lower-density systems such as TAS, the most profitable rootstocks were G.935, G.30, and B.118. Similarly, in the VA system, B.118 showed the highest profitability. These findings underscore the need to tailor rootstock selection to the specific training system maximizing long-term economic outcomes (**Figures 3**, **1**).

To better understand the evolution of cumulative NPV throughout the orchard’s lifespan, we plotted cumulative NPV from year 1 to year 20. As expected, all systems exhibited an initial decline in NPV during the first three years, reflecting the high establishment costs typically associated with planting and orchard development. After this initial period, all training systems and rootstocks showed a steady upward trend in cumulative NPV (**Figure 1**). Among the systems, VA experienced the smallest negative NPV during the early years, followed by TAS and then TS. In contrast, the SS system reached the most negative NPV in the first three years, likely due to its higher initial investment. However, both SS and TS systems demonstrated the fastest recovery, with a more rapid increase in cumulative NPV compared to the lower-density systems (TAS and VA), highlighting their greater long-term economic potential.

**Figure 4:**
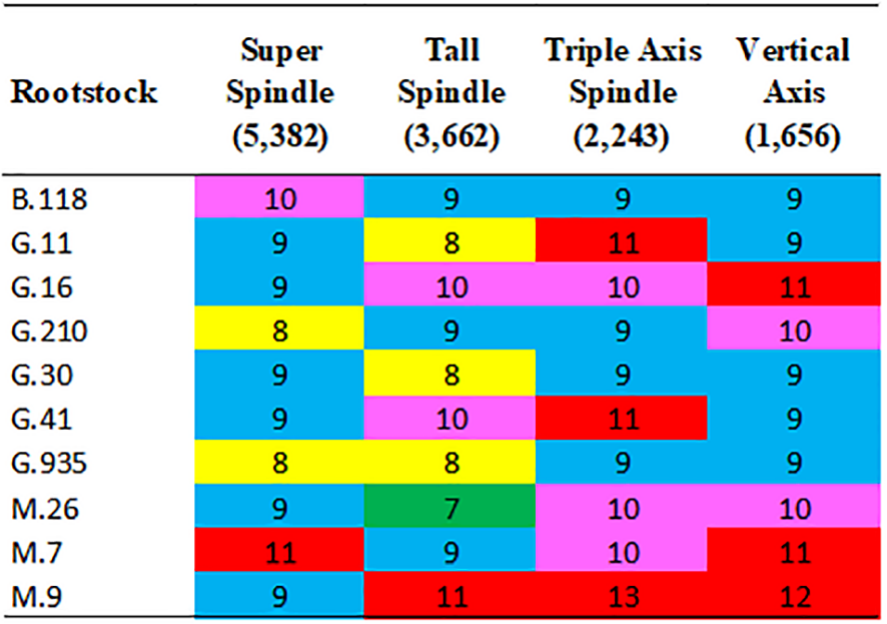
Break-even year for positive cumulative NPV from various combinations of training systems (in parentheses trees per hectare) and rootstocks with ‘Delicious’ grown in southeastern New York State. The training systems are arranged in order of decreasing tree planting density. Thus, with each rootstock, the effect of density can be observed by the change in break-even years between systems. Cells colored green and yellow had short break-even time period (7 and 8 years respectively), blue had intermediate break-even time periods (9 years) and pink (10 years), and red had the longest break-even time periods (>10 years).

The time required for cumulative profitability to reach the break-even point (NPV = 0) varied depending on the rootstock and training system (**Figures 1**, **4**). Across all combinations, the break-even year ranged from as early as year 7 to as late as year 13 (**Figure 4**). In the SS system, the break-even point was typically reached between years 8 and 11, depending on the rootstock. The fastest recovery was observed with G.210 and G.935, both reaching positive NPV in 8 years (yellow), indicating strong compatibility with high-density planting (**Figure 4**). Conversely, M.7 showed the poorest performance in this system, taking 11 years to reach profitability (red), likely because its high vigor and is low precocity which delays the onset of fruiting and significantly postpones the start of commercial production. Other dwarf and semi-dwarf rootstocks such as M.9, G.16, and G.30 cluster around 9 years, confirming that SS, despite its higher initial investment, had relatively quick returns when it was combined with suitable rootstocks (**Figure 4**). For the TS system, also a high-density system but with fewer trees per hectare and lower establishment costs than SS had the best overall performance. Break-even times range from 7 to 11 years, with M.26 achieving the shortest period at 7 years (green), making it the most efficient combination. Rootstocks like G.11, G.30, and G.935 follow closely at 8 years (yellow), reinforcing their adaptability to this system (**Figure 4**). In contrast, M.9 and G.41 required 11 years (red), suggesting that while they are compatible with high-density systems, their lower early productivity delayed profitability. In contrast, the lower-density systems (TAS and VA) tended to reach break-even later, between years 9 and 13 (**Figure 4**). These intermediate density systems exhibited significantly longer break-even periods, ranging from 9 to 13 years. No rootstock achieved green or yellow values with the lower density systems, reflecting slower economic recovery. The best rootstocks in TAS were B.118, G.210, G.30, and G.935, each reaching break-even in 9 years (blue) (**Figure 4**). However, M.9 showed the poorest performance with 13 years (red), followed by G.16 and G.41 at 11 years. These results indicate that TAS is less economically efficient in the short term. The lowest-density system, VA, consistently showed the longest break-even times, ranging from 9 to 12 years. Most rootstocks, including B.118, G.11, G.30, and G.935, were at 9 years (blue), suggesting moderate performance (**Figure 4**). However, dwarf rootstocks such as M.9 and G.16 were penalized, requiring 12 and 11 years respectively (red), due to their limited ability to generate early yields in low-density configurations. This pattern confirms that VA is the least economically attractive option, regardless of rootstock choice (**Figure 4**).

The training systems are arranged in order of decreasing tree planting density. Thus, with each rootstock, the effect of density can be observed by the change in break-even years between systems. Cells colored green and yellow had short break-even time period (7 and 8 years respectively), blue had intermediate break-even time periods (9 years) and pink (10 years), and red had the longest break-even time periods (>10 years).

### 3.2 Effect of tree density on NPV

**Figure 5:**
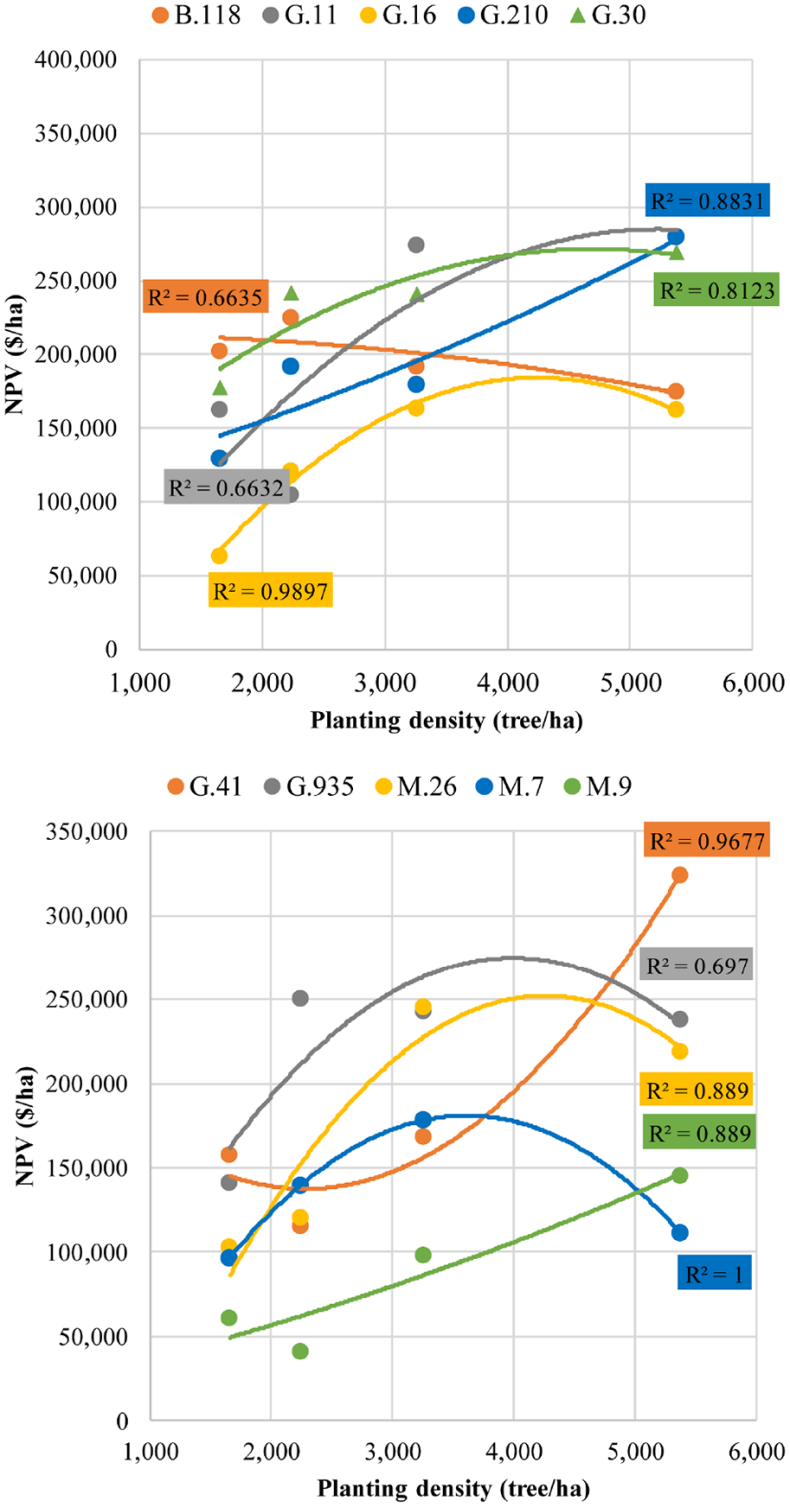
Quadratic regressions of tree planting density and cumulative net present value (NPV) for ‘Delicious’ and ten rootstocks trained to the four planting systems (Super Spindle = 5382 trees•ha^-1^, Tall Spindle = 3262 trees•ha^-1^, Triple Axis Spindle = 2243 trees•ha^-1^, and Vertical Axis 1656 trees•ha^-1^) in southeastern New York State. The rootstock curves were divided in two graphs soley to allow the reader to distinguish the curve for each rootstock.

**Figure 5** shows the regression between planting density (associated with training system) and NPV. The results show that each rootstock responds differently to changes in planting density. For G.11, G.210, G.30, G.41, and M.9, profitability increased as the number of trees per hectare increased, with the optimal density for these rootstocks being 5,382 trees/ha. However, G.41 exhibited a much steeper increase in NPV when density rose from 3,262 to 5,382 trees/ha, indicating that higher tree density is essential to maximize profitability for this rootstock (**Figure 5**). In contrast, the other rootstocks showed a less pronounced benefit from increasing density across all levels. Rootstocks G.16, G.935, M.26, and M.7 had their best profit at a density of 3,262 trees/ha. Beyond this threshold, 20-year profitability did not improve; in fact, for M.7, NPV decreased to levels similar to those observed at 1,656 trees/ha (**Figure 5**). B.118 was the only rootstock that exhibited a negative correlation with increasing tree density (**Figure 5**).

## 4 Discussion

This study is a continuation of the series of studies our group has conducted to determine the economics of varying planting density and rootstock with several varieties (Robinson et al., 2004; Lordan et al., 2018b; Gonzalez Nieto et al., 2023; Ho et al., 2024). This current study was done with a variety with a strong spur-type growth habit (Type 1) (Lespinasse, 1977) while all our previous studies were done with Type 3 varieties. Spur-type ‘Delicious’ has much shorter internodes and is lower in vigor than Type 3 varieties, thus, high-density plantings of spur-type ‘Delicious’ with M.9 have not been successful in commercial orchards. The unique growth habit of spur-type ‘Delicious’ presents challenges to producing spur-type ‘Delicious’ trees in modern high-density commercial orchards and is the reason this study was initiated.

The results of this study demonstrate that training system and rootstock play a crucial role in determining the long-term economic viability of ‘Delicious’ apple orchards, as assessed through NPV over a 20-year period. Among the systems we evaluated, the high-density planting configurations of SS and TS, consistently yielded the highest profitability. In contrast, the VA, characterized by its lower planting density, resulted in the least favorable economic returns, while the TAS occupied an intermediate position. These findings align with and expand upon previous research. For instance, Lordan et al. (2018b) demonstrated that the TS system provided optimal profitability for ‘McIntosh’ apples at a similar planting density, although their study did not include the SS system. The agronomic analysis by Reig et al. (2020), based on the same ‘Delicious’ dataset, showed that SS had the highest cumulative yields, followed by TS, with TAS and VA trailing behind. This yield hierarchy directly correlates with the economic outcomes observed in our study, reinforcing the conclusion that systems with higher cumulative yields tend to generate greater long-term profits. Our data further supports the conclusions from Mitre et al. (2011), who found that TS outperformed VA and Solaxe in terms of productivity across various apple cultivars. Our findings with ‘Delicious’, clearly show that TS and SS are economically superior due to their higher yield potential. Additionally, Weber (1998) compared SS with the traditional Slender Spindle system and concluded that although SS required a higher initial investment, it delivered significantly greater profits after a decade. Our study extends this observation over a 20-year horizon, confirming that despite the elevated establishment costs associated with high-density systems like SS and TS, their long-term profitability is substantially higher.

Our results with the Triple Axis Spindle system indicate that lowering the number of trees per acre and developing multiple leaders (trunks) on each tree resulted in lower cumulative NPV than single stem trees of TS or SS both planted at higher densities than the TAS. The planting density of the TAS was 2,343 trees per hectare resulting in 6,729 leaders per hectare which was greater than the SS which had 5,382 leader (trunks) per hectare or the TS which had 3,662 trees per hectare yet the TAS had lower profitability. Thus, our results indicate that it is a false promise that a multi leader tree will allow a reduction in the number of trees per hectare resulting in similar profitability as higher density single stem trees. Our result also indicates that the idea of developing more leaders per tree to allow lower planting densities to reduce the initial establishment costs of the high density systems such as TS or SS systems, negatively affects lifetime profitability. Underlying this result is the basic principle that profitability is strongly tied to planting density up to 3,000-5,000 trees per hectare as we have previously reported (Lordan et al., 2019b). The curvilinear profitability curve we previously reported for 4 Type 3 cultivars showed profitability maximized at around 3,000 trees per hectare. The current study with spur-type ‘Delicious’ indicates that the optimum density (depending on rootstock) is probably ~4,000 trees per hectare.

There was a significant interaction between rootstock and training system, indicating that the economic performance of a given rootstock varied depending on the training system, which supports findings previously reported by Ho et al. (2024) and Gonzalez Nieto et al. (2024), who observed similar interaction between training system, rootstock and variety. This finding showed that profitability cannot be optimized by considering rootstock or training system in isolation. For example, while M.9 consistently ranked among the least profitable rootstocks overall, its slow early growth, despite being a precocious rootstock, limits early yields; however, it showed greater profit under SS than under VA, suggesting that high-density systems can partially offset this disadvantage. In this line, Lawrence et al. (2025) reported similar results for ‘Delicious’ with M.9T337 where this rootstock was among the least productive in a 17-year trial comparing 18 rootstocks, further confirming its limited economic potential in long-term orchard systems. Interestingly, M.7 had the poorest results with SS despite performing moderately in lower-density systems, highlighting that vigorous rootstocks that are non-precocious may not align well with high-density plantings. Cline et al. (2025) had similar results with ‘Honeycrisp’ in a multilocation trial in the US. In our study, G.11 and G.41 emerged as top performers with the SS system, whereas G.11 was the best with TS, and B.118 provided the highest returns in VA, reflecting the adaptability of the different rootstocks to specific structural and management conditions. These patterns reveal that rootstock traits such as vigor and precocity interact strongly with system design, influencing the profitability of the orchard, as mentioned by Fazio (2021). This author noted that the recent advances in apple breeding programs have greatly enhanced the capacity to tailor rootstock performance to specific orchard conditions, including scion cultivar, soil characteristics, water availability, disease pressure, and training system architecture. Therefore, economic sustainability in apple orchards depends on a tailored approach that matches rootstock characteristics with the chosen training system, rather than relying on general recommendations. This interaction of training system and rootstock genotype highlights the complexity of orchard design decisions and reinforces the need for integrated strategies that consider both biological and economic factors to maximize long-term profitability.

In our study, large differences in establishment costs were associated with each training systems, as shown in previous work (Robinson et al., 2004; Lordan et al., 2018b; Gonzalez Nieto et al., 2023; Ho et al., 2024) and several other papers. This early negative balance with all systems reflects the implementation costs of orchard establishment. VA showed the smallest initial deficit, followed by TAS and TS, while SS experienced the most negative NPV during the early years. This is logical because the most intensive apple orchards had higher implementation cost because the number of trees, and materials (irrigations lines, post, wires, etc.) was higher for high density systems compared to low density systems. However, this initial disadvantage was offset by greater early yield of high-density systems leading to a rapid recovery of cumulative NPV observed in SS and TS, which demonstrated the steepest upward trajectory in cumulative NPV after the establishment phase. This result coincides with the results of Weber (1998). This pattern of the more rapid improvement of cumulative NPV’s highlights the economic advantage of high-density systems, where early and sustained yield compensates for higher upfront costs, ultimately leading to superior long-term profitability. In contrast, lower-density systems such as TAS and VA exhibited slower NPV recovery rates, reflecting their reduced capacity for early production and lower cumulative yields. These results are consistent with the findings of Quamme et al. (1996) who showed how the high-density systems like slender spindle and VA are more productive and economically beneficial than low density systems especially in the early years of orchard establishment, despite slightly higher labor demands. These findings reinforce the principle that orchard profitability is strongly influenced by the balance between initial investment and the speed of revenue generation, favoring high-density systems for long-term economic sustainability.

Among rootstocks, the SS system with rootstocks like G.210 and G.935 reached break-even in just 8 years, confirming their strong compatibility with intensive planting. Conversely, M.7 showed the slowest recovery, requiring 11 years, likely due to its vigor and delayed fruiting. With the TS system, the fastest break-even was with M.26 in 7 years. However, G.11, G.30, and G.935 need 8 years, reinforcing their adaptability to high-density configurations. M.9 and G.41 required longer recovery times, suggesting that despite their compatibility, their lower early productivity can delay profitability. Lordan et al. (2018b) concluded that the faster break-even with M.9 for ‘McIntosh’ (9 years) and B.9 and M.9 for ‘Honeycrisp’ (7 and 8 years respectively). Their conclusion with ‘McIntosh’ and ‘Honeycrisp’ was different from our results with M.9. This was likely due to the extremely low vigor of ‘Delicious’ with M.9 which resulted in small trees that did not fully fill the space as with the varieties in the Lordan et al. (2018b) study. This result supports the concept of matching scion vigor with a precocious rootstock that will fill the allotted space rapidly.

The lower density systems, TAS and VA showed significantly longer break-even periods, ranging from 9 to 13 years. Bechtel et al. (1995) reported a break-even year of ‘Delicious’ with central leader and M.7 and M.26 between 11 and 14 years in Washington state. Our results with TAS showed that more vigorous rootstocks such as B.118, G.210, G.30, and G.935 reached break-even in 9 years, while M.9 performed poorly, requiring 13 years. The lowest density system we evaluated was VA which consistently showed the longest break-even times, with dwarf rootstocks like M.9 and G.16 penalized due to limited early yield potential.

The regression analysis between planting density, as determined by the training system, and cumulative NPV over 20 years revealed that economic performance is improved by increasing planting density up to an optimum density which varies considerably among rootstocks. B.118 was the only rootstock that showed a negative correlation between planting density and NPV, with the highest profitability observed at the lowest density evaluated (1,656 trees/ha). This negative relationship was likely due to excessive vigor of this stock which required excessive pruning in the high density systems. The excessive pruning was counterproductive to fruit production. These findings are consistent with those reported by Lo Bianco et al. (2003), who concluded that apple tree growth is influenced by both rootstock vigor and tree spacing. Their study demonstrated that the same rootstock exhibited different growth patterns depending on the planting density, which aligns with our results indicating that each rootstock has a specific optimal tree planting density that maximizes productivity and profitability.

While this study did not evaluate the effect of rootstock and training system on fruit size, external and internal quality, and storability other studies have reported rootstock effects on fruit size (Lawrence et al., 2025) and on fruit quality (Al Shoffe et al., 2025; Gonzalez Nieto et al., 2025; Valverdi and Kalcsits, 2021). It is likely that the rootstocks we evaluated in this study, had important effects on fruit size and fruit quality. However, those effects are unlikely to have a significant effect on orchard profitability which is largely driven by price and yield. Fruit size can have an important effect on crop value but it is largely controlled by crop load (Gonzalez Nieto et al., 2025). We actively managed crop load in this study so that each year each system and rootstock had a commercially acceptable crop load, which minimizes differences in fruit size.

Recognizing the limitations of this study (recorded data only through year 11 and estimated data of yield for years 12–20 and the lack of data on fruit quality and size), this study is the first study in ‘Delicious’ apples to evaluate the interaction between rootstock and tree density from an economic perspective. Our results suggest that for rootstocks such as G.11, G.210, G.30, G.41, and M.9, profitability was positively related to planting density with highest planting density producing the greatest profitability, particularly for G.41. This indicates that high-density planting is critical to achieving the maximum economic potential for this rootstock. Ho et al. (2024) reached similar conclusions in a comparable analysis, reporting that the optimal planting density ranged between 2,500 and 3,000 trees per hectare for M.9, G.41, and G.16 in ‘Fuji’, ‘Honeycrisp’, and ‘Gala’. However, for ‘Fuji’ grafted on G.41, the optimal density was below 1,000 trees/ha, highlighting that the same rootstock may perform differently depending on the scion cultivar. In our study, B.118 was the only rootstock which performed best at the lowest density evaluated (1,656 trees/ha). Meanwhile, G.16, G.935, M.26, and M.7 achieved their highest economic returns at an intermediate density of 3,262 trees/ha in the TS system, with no additional gains observed beyond this threshold over a 20-year period. No previous studies have directly examined the relationship between tree spacing and economic performance of ‘Delicious’ for these Geneva rootstocks.

## 5 Conclusions

This study provides the first comprehensive economic evaluation of the interaction between rootstock and planting density with spur-type ‘Delicious’ apple orchards, using NPV over a 20-year period. The results demonstrate that both the training system and rootstock significantly influence long-term profitability, and that their interaction must be considered when designing economically sustainable orchard systems.

High-density training systems, particularly SS and TS, consistently outperformed lower-density systems in terms of profitability, despite higher initial establishment costs. These systems had faster break-even points and greater cumulative NPV, especially when combined with rootstocks such as G.11, G.210, and G.935. In contrast, Vertical Axis (VA) and Triple Axis Spindle (TAS) systems exhibited slower economic recovery and lower overall returns.

Rootstock profitability was highly dependent on planting density and training system. G.41, G.11, and G.210 showed strong economic performance under high-density systems, while economic performance of B.118 was optimum at lower densities. However, M.9 and M.7 demonstrated limited economic potential with spur-type ‘Delicious’, particularly for intensive systems. These findings confirm that rootstock vigor and precocity must be aligned with training system and scion variety vigor to optimize orchard profitability.

The study shows that orchard design should be adapted to the unique conditions of each orchard. Rather than relying on generalized recommendations, growers should consider the specific interactions between rootstock, scion cultivar, training system, and site conditions. This integrated strategy is essential for maximizing long-term economic returns and sustainability of modern apple production systems.

## Data Availability

The raw data supporting the conclusions of this article will be made available by the authors, without undue reservation.
